# Time-ordering in Heisenberg’s equation of motion as related to spontaneous radiation

**DOI:** 10.1038/s41598-021-01781-7

**Published:** 2021-11-12

**Authors:** Benjamin D. Strycker

**Affiliations:** 1 Baylor Research and Innovation Collaborative, Baylor University, Waco, TX 76704 USA; 2 Institute for Quantum Science and Engineering, Texas A&M University, College Station, TX 77843 USA

**Keywords:** Quantum optics, Theoretical physics

## Abstract

Despite many years of research into Raman phenomena, the problem of how to include both spontaneous and stimulated Raman scattering into a unified set of partial differential equations persists. The issue is solved by formulating the quantum dynamics in the Heisenberg picture with a rigorous accounting for both time- and normal-ordering of the operators. It is shown how this can be done in a simple, straightforward way. Firstly, the technique is applied to a two-level Raman system, and comparison of analytical and numerical results verifies the approach. A connection to a fully time-dependent Langevin operator method is made for the spontaneous initiation of stimulated Raman scattering. Secondly, the technique is demonstrated for the much-studied two-level atom both in vacuum and in a lossy dielectric medium. It is shown to be fully consistent with accepted theories: using the rotating wave approximation, the Einstein *A* coefficient for the rate of spontaneous emission from a two-level atom can be derived in a manner parallel to the Weisskopf–Wigner approximation. The Lamb frequency shift is also calculated. It is shown throughout that field operators corresponding to spontaneous radiative terms do not commute with atomic/molecular operators. The approach may prove useful in many areas, including modeling the propagation of next-generation high-energy, high-intensity ultrafast laser pulses as well as spontaneous radiative processes in lossy media.

## Introduction

The question of how to account for spontaneous radiation is closely connected with the foundations of quantum optics. It was this question that first led Einstein in 1917 away from a classical, isotropic model of radiation emission and toward a highly-directional, quantum-mechanical model characterized by the well-known *A* and *B* coefficients of spontaneous and stimulated emission^1^. Ten years later Dirac derived the forms of Einstein’s *A* and *B* coefficients using the new quantum theory developed by Schrödinger and Heisenberg, simultaneously postulating the quantization of the electromagnetic field as a collection of harmonic oscillators for the first time^2,3^. It was found that the harmonic oscillator model was sufficient to explain the light-matter interaction, and it subsequently became a key feature of quantum optics theory.

In the following years, opinions wavered over whether spontaneous radiation resulted primarily from the fluctuations of the quantized electromagnetic field or from the quantum analogue of the classical radiation reaction. In 1963 Jaynes and Cummings showed that spontaneous emission could result from a model in which the fields remain classical and the radiating electron experiences radiation reaction^4^. Ultimately, this model was found to give clearly spurious results for anti-Stokes Raman scattering^5^, since in a semi-classical model the spontaneous transition rate depends on both the initial and final states, while for a fully quantum model the transition rate depends only on the initial state^5–7^. In addition, it had been known since 1958 that semi-classical radiation theory can only account for the coherent aspect of spontaneous emission and neglects the incoherent contribution^7–9^. In 1973, Senitzky brought the controversy between vacuum fluctuations and radiation reaction to a satisfactory conclusion when he showed that their roles in spontaneous emission are “two sides of the same quantum-mechanical coin". Each phenomenological picture may be brought into clearer focus through different orderings of the relevant quantum operators^10^. Vacuum fluctuations and radiation reaction are therefore related through a fluctuation-dissipation theorem^11^. This revelation, however, does not mean that further insight cannot be gained from semi-classical models of spontaneous radiation^12^.

Even as the theory of Raman scattering was playing a minor role in the elucidation of the nature of spontaneous radiation processes in general, research into Raman phenomena in their own right was developing apace. Placzek had detailed the quantum theory of spontaneous Raman scattering in 1934^13^, but, with the invention of the laser, focus soon shifted to stimulated Raman processes. Beginning with Shen and Bloembergen in 1965, the theory of stimulated Raman scattering in both the forward and backward directions was formulated^14–17^. These works used both classical and semi-classical models to account for pulse propagation effects. As such, the effects of spontaneous Raman scattering were neglected. The first attempts to unify both spontaneous and stimulated Raman scattering within a single theoretical chassis accounting for pulse propagation were undertaken by Raymer and Mostowski in a pair of papers published in 1981^18,19^. Their work assumed a heavily populated ground state and introduced a quantum statistical Langevin operator corresponding to “collision-induced fluctuations” that was shown to reproduce predicted behavior in both the spontaneous and stimulated Raman regimes. This kind of approach is at best incomplete, since it is known both experimentally and quantum-theoretically that, while collisions do induce Raman coherences^20^, an isolated molecule may spontaneously scatter Raman radiation independently of collisions. In subsequent work investigating macroscopic fluctuations in the transient Raman regime^21–25^, they make a distinction between spontaneous Raman radiation induced by vacuum fluctuations and by collisions, and continue to use the Langevin operator as a phenomenological tool.

The limitations of this kind of model immediately become apparent as soon as the assumption of a heavily populated ground state is relaxed, as in the case of laser pulses of sufficient intensity to induce a significant fraction of molecules into a vibrationally excited state. Such considerations are especially important in the development of stimulated Raman backscattering amplification (SRBA) of high-energy, high-intensity ultrafast laser pulses, which is regarded as a next-generation technology to surpass the intensity limitations of chirped pulse amplification (CPA). SRBA is envisioned to possibly enable exawatt and even zettawatt laser powers^26^, but two decades of effort in this area has yielded little success^27^.

For laser pulses with this level of intensity, experiment indicates that the interplay between spontaneous and stimulated Raman radiation becomes vitally important. What kind of theoretical model can accurately describe this system? A semi-classical approach readily accommodates the dynamics of pulse propagation but fails to describe the all-important contribution from spontaneous Raman processes in a satisfactory manner. Only a fully quantum-mechanical model, in which the fields are also quantized, will be able to account for the diversity of behavior. Yet, using a conventional Raman Hamiltonian in Heisenberg’s equation of motion yields only coherent, stimulated terms.

Spontaneous Raman processes are typically described in the interaction picture through calculation of transition probabilities using the Dyson U-matrix operator^28^. Specifically, the spontaneous terms result from normal-ordering of field operators within a perturbative time-ordered theoretical framework. The dynamics are usually solved to a few orders of the perturbation, meaning that the solution is valid only for short times. Complete knowledge of the behavior’s system for all time requires calculation of an infinite number of perturbative orders.

Such prohibitively arduous calculations can be circumvented through incorporating both time- and normal-ordering of operators into Heisenberg’s equation of motion, from which the approximate, time-ordered perturbative methods were originally derived. In order to do so, it is necessary to point out an insight originally discovered by Feynman^29^ that commutators arise at least in part from the chronological order of the operators involved. In other words, for two canonically conjugate operators ${\hat{p}}$ and ${\hat{q}},$

1 $$\begin{aligned} {}[{\hat{p}},{\hat{q}}] = {\hat{p}}_{k+1} {\hat{q}}_k - {\hat{q}}_k {\hat{p}}_{k-1} = -i \hbar , \end{aligned}$$

or, in the case of creation and annihilation operators ${\hat{a}}^{\dagger }$ and ${\hat{a}},$

2 $$\begin{aligned} {}[{\hat{a}},{\hat{a}}^{\dagger }] = {\hat{a}}_{k+1} {\hat{a}}_k^{\dagger } - {\hat{a}}_k^{\dagger } {\hat{a}}_{k-1} = 1 , \end{aligned}$$

where in Eqs. (1) and (2) the indices correspond to time-ordered infinitesimal time intervals. It should be noted that the indices need not be sequential; they need only to be in order, with greater values occurring later (to the left) of earlier values (placed to the right). In addition, time indices play a role only for non-commuting operators^30^.

Heisenberg’s equation of motion for an operator ${\hat{A}}$ may also be written as an explicit time-ordered expression^29^:

3 $$\begin{aligned} \frac{d {\hat{A}}}{dt} = \frac{i}{\hbar }[{\hat{H}},{\hat{A}}] = {\hat{H}}_{k+1} {\hat{A}}_k - {\hat{A}}_k {\hat{H}}_{k-1}. \end{aligned}$$

In a system of differential equations for non-commuting operators, the correct time-ordering of the operators cannot be neglected when substitutions are made in the course of evaluation. This is shown first for a two-level Raman system. Then, to demonstrate the robust validity of the approach, it is demonstrated for the much-studied two-level atomic system, both in vacuum and in a lossy dielectric.

## Results

### The two-level Raman system

Here, the technique is demonstrated for spontaneous Raman scattering from a two-level molecular system defined by a ground state ${|}{b}{\rangle }$ and an excited vibrational state ${|}{a}{\rangle }$, with molecular operators $\hat{\tilde{\sigma }}$ that obey the Heisenberg equation of motion and are defined by

4 $$\begin{aligned} \hat{\tilde{\sigma }}_{nm}&= {|}{n}{\rangle }{\langle }{m}{|} \end{aligned}$$

5 $$\begin{aligned} \hat{\tilde{\sigma }}_{ij} \hat{\tilde{\sigma }}_{kl}&= \hat{\tilde{\sigma }}_{il} \, \delta _{jk}. \end{aligned}$$

The system is governed by an unperturbed molecular Hamiltonian ${\hat{H}}_M,$ a Raman interaction Hamiltonian ${\hat{H}}_I,$ and a field Hamiltonian ${\hat{H}}_F$ such that

6 $$\begin{aligned} {\hat{H}} = {\hat{H}}_M + {\hat{H}}_I + {\hat{H}}_F  , \end{aligned}$$

where

7 $$\begin{aligned} {\hat{H}}_M&= \hbar \omega _a \, {\hat{\sigma }}_{aa} + \hbar \omega _b \, {\hat{\sigma }}_{bb} \end{aligned}$$

8 $$\begin{aligned} {\hat{H}}_I&= -G \, \hat{{\tilde{a}}}_p \hat{{\tilde{a}}}_s^{\dagger } \, \hat{\tilde{\sigma }}_{ab} + \text {adj.} \end{aligned}$$

9 $$\begin{aligned} {\hat{H}}_F&= \sum _k \hbar \omega _k \left[ \hat{{\tilde{a}}}_k^{\dagger } \hat{{\tilde{a}}}_k + \frac{1}{2} \right] , \end{aligned}$$

and the coupling factor *G* is given by

10 $$\begin{aligned} \begin{aligned} G = \frac{1}{2\hbar }\sqrt{\frac{\hbar \omega _p}{\epsilon _0 V}}\sqrt{\frac{\hbar \omega _s}{\epsilon _0 V}} \sum _{i} \left[ \frac{ {\langle }{a}{|} \varvec{\mu }_{ai} \cdot \varvec{\epsilon }_s {|}{i}{\rangle } {\langle }{i}{|} \varvec{\mu }_{ib} \cdot \varvec{\epsilon }_p {|}{b}{\rangle } }{\omega _{ib}-\omega _p} \right. + \left. \frac{ {\langle }{a}{|} \varvec{\mu }_{ai} \cdot \varvec{\epsilon }_p {|}{i}{\rangle } {\langle }{i}{|} \varvec{\mu }_{ib} \cdot \varvec{\epsilon }_s {|}{b}{\rangle } }{\omega _{ib}+\omega _s} \right] , \end{aligned} \end{aligned}$$

where $\varvec{\mu }_{ij} = e {\mathbf{r}}_{ij}$ is the dipole moment between levels ${|}{i}{\rangle }$ and ${|}{j}{\rangle},$ $\varvec{\epsilon }_{{\mathbf{k}}}$ is the polarization vector of field mode $\mathbf{k},$ and *p* and *s* designate the pump and Stokes fields, respectively.

Taking into account the time-ordering aspects of the Heisenberg equation of motion as outlined above (and using overset indices to indicate time-order), the equations of motion for the molecule and field are then

11 $$\begin{aligned} \dot{{\hat{\sigma }}}_{ab}&= -i \frac{ G }{\hbar } e^{-i \Delta t} \left[ \overset{2}{\overline{{\hat{a}}_p^{\dagger } {\hat{a}}_s \, {\hat{\sigma }}_{bb} }} - \overset{1}{ \overline{ {\hat{a}}_p^{\dagger } {\hat{a}}_s \, {\hat{\sigma }}_{aa} }} \right] \end{aligned}$$

12 $$\begin{aligned} \dot{{\hat{\sigma }}}_{ba}&= -i \frac{ G }{\hbar } e^{i \Delta t} \left[ \overset{2}{\overline{{\hat{a}}_p {\hat{a}}^{\dagger }_s \, {\hat{\sigma }}_{aa} }} - \overset{1}{ \overline{ {\hat{a}}_p {\hat{a}}^{\dagger }_s \, {\hat{\sigma }}_{bb} }} \right] \end{aligned}$$

13 $$\begin{aligned} \dot{{\hat{\sigma }}}_{aa}&= \dot{\hat{a^{\dagger }_s a_s}} = -i \frac{ G }{\hbar } \left[ e^{-i \Delta t} \, \overset{2}{ \overline{ {\hat{a}}_p^{\dagger } {\hat{a}}_s \, {\hat{\sigma }}_{ba} }} - e^{i \Delta t} \, \overset{1}{ \overline{ {\hat{a}}_p {\hat{a}}_s^{\dagger } \, {\hat{\sigma }}_{ab} }} \right] \end{aligned}$$

14 $$\begin{aligned} \dot{{\hat{\sigma }}}_{bb}&= \dot{\hat{a^{\dagger }_p a_p}} = -i \frac{ G }{\hbar } \left[ e^{i \Delta t} \, \overset{2}{ \overline{ {\hat{a}}_p {\hat{a}}_s^{\dagger } \, {\hat{\sigma }}_{ab} }} - e^{-i \Delta t} \, \overset{1}{ \overline{ {\hat{a}}_p^{\dagger } {\hat{a}}_s \, {\hat{\sigma }}_{ba} }} \right] \end{aligned}$$

15 $$\begin{aligned} \dot{{\hat{a}}}_p&= \left( \dot{{\hat{a}}}_p^{\dagger } \right) ^{\dagger } = i \frac{G}{\hbar } e^{-i \Delta t} \, \overset{1}{ \overline{ {\hat{a}}_s \, {\hat{\sigma }}_{ba} }} \end{aligned}$$

16 $$\begin{aligned} \dot{{\hat{a}}}_s&= \left( \dot{{\hat{a}}}_s^{\dagger } \right) ^{\dagger } = i \frac{G}{\hbar } e^{i \Delta t} \, \overset{1}{\overline{ {\hat{a}}_p \, {\hat{\sigma }}_{ab} }} , \end{aligned}$$

where ${\hat{\sigma }}_{ij} = \hat{\tilde{\sigma }}_{ij} \, e^{-i \, \omega _{ij} t}$, ${\hat{a}}_{{\mathbf{k}}} = \hat{{\tilde{a}}}_{{\mathbf{k}}} \, e^{i \, \omega _{{\mathbf{k}}} t}$, and $\Delta \equiv \omega _{ab} - (\omega _p - \omega _s)$ is the two-photon detuning. Eqs. (11)–(16) introduce the convention of initially placing the molecular operators to the right of the field operators.

The time- and normal-ordered solution of these equations can be illustrated in a straightforward manner. For example, the operator ${\hat{\sigma }}_{aa}$ can be written as the sum of two functions, each operating in single time bin only such that

17 $$\begin{aligned} {\hat{\sigma }}_{aa}&= {\hat{\sigma }}^{(2)}_{aa} + {\hat{\sigma }}^{(1)}_{aa} \end{aligned}$$

18 $$\begin{aligned} \dot{{\hat{\sigma }}}^{(2)}_{aa}&= -i \frac{ G }{\hbar } e^{-i \Delta t} \, \overset{2}{ \overline{ {\hat{a}}_p^{\dagger } {\hat{a}}_s \, {\hat{\sigma }}_{ba} }} \end{aligned}$$

19 $$\begin{aligned} \dot{{\hat{\sigma }}}^{(1)}_{aa}&= i \frac{ G }{\hbar } e^{i \Delta t} \, \overset{1}{ \overline{ {\hat{a}}_p {\hat{a}}_s^{\dagger } \, {\hat{\sigma }}_{ab} }} . \end{aligned}$$

Allowing the other operators to be expressed in the same manner yields, for $\dot{{\hat{\sigma }}}^{(2)}_{aa},$

20 $$\begin{aligned} \dot{{\hat{\sigma }}}^{(2)}_{aa}&= -i \frac{ G }{\hbar } e^{-i \Delta t} \, \overset{2}{ {\hat{a}}_p^{\dagger } } \overset{2}{ {\hat{a}}_s } \, \left[ \overset{3}{ {\hat{\sigma }}^{(2)}_{ba} } + \overset{2}{ {\hat{\sigma }}^{(1)}_{ba} } \right] \end{aligned}$$

21 $$\begin{aligned}&= -i \frac{ G }{\hbar } e^{-i \Delta t} \, \left[ \overset{3}{ {\hat{\sigma }}^{(2)}_{ba} } \, \overset{2}{ {\hat{a}}_p^{\dagger } } \overset{2}{ {\hat{a}}_s } + \overset{2}{ {\hat{a}}_p^{\dagger } } \overset{2}{ {\hat{a}}_s } \, \overset{2}{ {\hat{\sigma }}^{(1)}_{ba} } \right] \end{aligned}$$

22 $$\begin{aligned}&= -i \frac{ G }{\hbar } e^{-i \Delta t} \, \left[ \left( {\hat{a}}_p^{\dagger } \, {\hat{\sigma }}^{(2)}_{ba} + \frac{\partial \, {\hat{\sigma }}^{(2)}_{ba} }{ \partial \, {\hat{a}}_p } \right) \, {\hat{a}}_s \right. + \left. {\hat{a}}_p^{\dagger } \left( {\hat{\sigma }}^{(1)}_{ba} \, {\hat{a}}_s + \frac{\partial \, {\hat{\sigma }}^{(1)}_{ba} }{ \partial \, {\hat{a}}^{\dagger }_s } \right) \right] . \end{aligned}$$

In going from Eqs. (21) and (22), it is assumed that the molecular operators are normal-ordered functions of the field operators. In Eq. (21), the operators are time-ordered but not normal-ordered, while in Eq. (22) the commutation relations

23 $$\begin{aligned} \left[ {\hat{a}}_{{\mathbf{k}}}, f({\hat{a}}^{\dagger }_{{\mathbf{k}}}) \right]&= \frac{ \partial \, f({\hat{a}}^{\dagger }_{{\mathbf{k}}}) }{\partial \, {\hat{a}}^{ \dagger }_{{\mathbf{k}}} } \end{aligned}$$

24 $$\begin{aligned} \left[ {\hat{a}}^{\dagger }_{{\mathbf{k}}}, f({\hat{a}}_{{\mathbf{k}}}) \right]&= - \frac{ \partial \, f({\hat{a}}_{{\mathbf{k}}}) }{\partial \, {\hat{a}}_{{\mathbf{k}}} } \, \end{aligned}$$

have been used. Since in Eq. (22) the operators are both time- and normal-ordered, the time indices may be omitted. The partial derivative terms may be evaluated through simple integration of Eqs. (11)–(16), and, although implicit dependence on the field operators may be accounted for, it is shown below that differentiation with respect to explicit field operator dependence is sufficient to achieve accurate results.

For example, an analytical perturbative solution in powers of a parameter $\lambda$ can be found if, for any operator ${\hat{A}}$ and the coupling constant *G*, the substitutions

25 $$\begin{aligned} {\hat{A}}&\rightarrow {\hat{A}}^{(0)} + \lambda \, {\hat{A}}^{(1)} + \lambda ^2 \, {\hat{A}}^{(2)} + \cdots \end{aligned}$$

26 $$\begin{aligned} G&\rightarrow \lambda G, \end{aligned}$$

are made. In this case, using the procedure outlined above and the initial conditions ${\hat{\sigma }}_{aa}(0) = 0$, ${\hat{\sigma }}_{bb}(0) = 1$, it is straightforward to show that up to second order in $\lambda$

27 $$\begin{aligned} \langle {\hat{\sigma }}_{aa} \rangle = \frac{G^2}{\hbar ^2} \, n_p \left( n_s +1 \right) \frac{ \sin ^2(\frac{\Delta }{2} t)}{\left( \frac{\Delta }{2} \right) ^2} , \end{aligned}$$

where $n_p$ and $n_s$ are the number of pump and Stokes photons, respectively. Equation (27) is the probability of finding the molecule in the excited vibrational state ${|}{a}{\rangle }$ as a function of time. It is identically equal to the probability of a spontaneous Raman transition from state ${|}{b}{\rangle }$ to ${|}{a}{\rangle }$ as calculated with the conventional U-matrix method up to second order using the conventional interaction Hamiltonian ${\hat{V}} = - \hat{\varvec{\mu }} \cdot \hat{{\mathbf {E}}}$.

**Figure 1:**
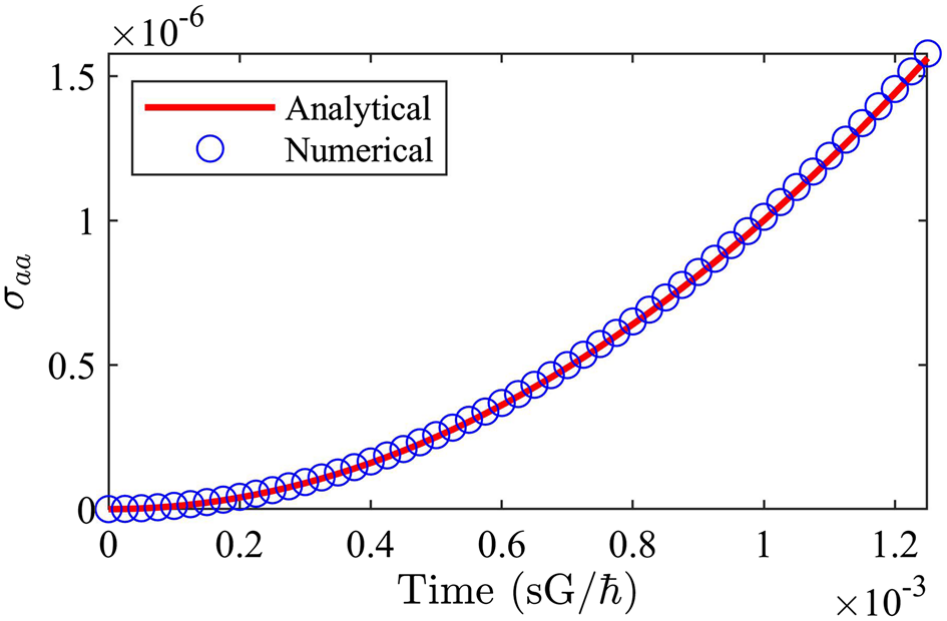
Comparison of a perturbative analytical solution (according to Eq. (27)) and a numerical solution (calculated according to time- and normal-ordered Eqs. (11)–(16)) for the excited vibrational state population ${\hat{\sigma }}_{aa}$ for small times. Here, the two-photon detuning $\Delta =0$.

**Figure 2:**
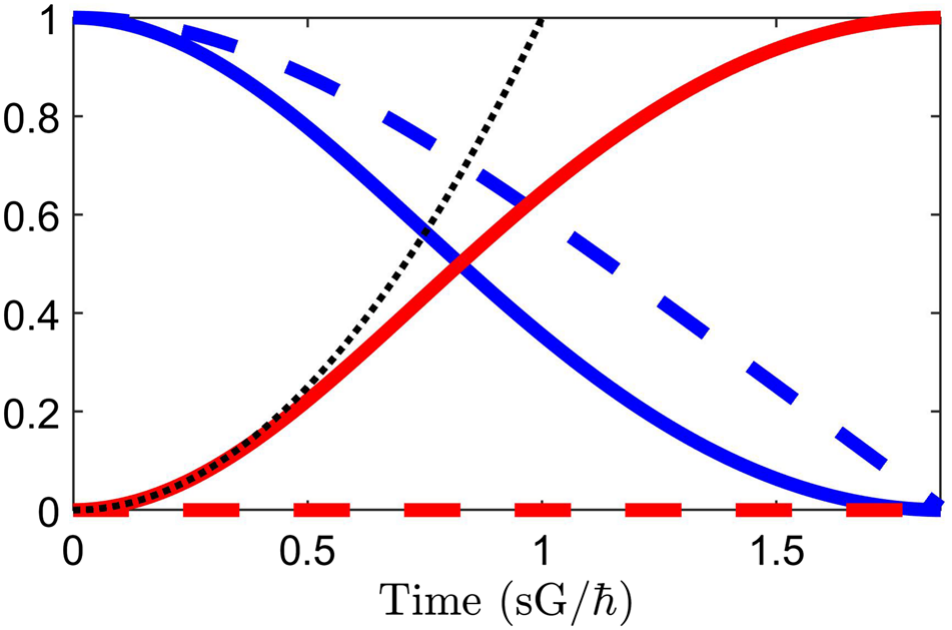
Full evolution of operators in a one-photon spontaneous Raman scattering process. Blue curves correspond to pump operators, while red curves correspond to Stokes operators. Solid curves correspond to intensities $\hat{a^{\dagger }_{{\mathbf{k}}} a_{{\mathbf{k}}} }$ , while dotted curves correspond to fields ${\hat{a}}_{{\mathbf{k}}}$. Curves corresponding to ${\hat{\sigma }}_{bb}$ and ${\hat{\sigma }}_{aa}$ follows those of $\hat{a^{\dagger }_p a_p }$ and $\hat{a^{\dagger }_s a_s }$, respectively. The dotted black curve corresponds to the perturbative analytical solution for ${\hat{\sigma }}_{aa}$ in Eq. (27). Here, the two-photon detuning $\Delta =0$.

Again, such a perturbative solution is accurate only for small times. As the time increases beyond the domain of the perturbation, a numerical calculation using time- and normal-ordered Eqs. (11)–(16) yields greater accuracy, since in fact it takes account of all perturbative orders. This kind of numerical solution (conventional 4th order Runge–Kutta, and where the two-photon detuning $\Delta =0$) is shown in Figs. 1 and  2 for the case of a single pump photon scattering from a molecule and spontaneously generating a Stokes photon. Figure 1 shows that, for small times, Eqs. (11)–(16) are identical to the analytical perturbative solution in Eq. (27) when solved according to the method outlined in Eqs. (20)–(22). Figure 2 shows the numerical solution for large times, beyond the domain of the analytical perturbative solution in Eq. (27). In particular, the analytical solution for ${\hat{\sigma }}_{aa}$ in Eq. (27) (dotted black line) is parabolic for all *t* given the Hamiltonian in Eqs. (7)–(9), while the numerical solution for ${\hat{\sigma }}_{aa}$ according to time- and normal-ordered Eqs. (11)–(16) (solid red line) approaches unity as time progresses, for the same Hamiltonian. The end result is that the pump photon is annihilated, a Stokes photon is created, and the molecule has transitioned to the excited vibrational state. These results clearly show the advantage of the approach outlined above when the value of *t* increases beyond perturbative domains.

Of interest in Fig. 2 is the fact while the Stokes intensity increases by one photon, the Stokes field remains at zero. Physically, this corresponds to the unpredictability of the phase of a spontaneously generated photon. Spontaneous Raman scattering is an incoherent process. If, alternatively, the incoming Stokes field were nonzero, the scattering process would contain both coherent and incoherent components, resulting in a corresponding change in the Stokes field amplitude. As they stand, Eqs. (11)–(16), when both time- and normal-ordered, model stimulated (coherent) and spontaneous (incoherent) scattering processes separately. In order to model spontaneously initiated stimulated Raman scattering, an additional equation is required to model the stochastic manner in which incoherent, spontaneously generated Stokes photons contribute to coherent processes.

This is generally done using a Langevin operator formalism^19,31^. The quantum-mechanical formulation of the Langevin approach is described in detail in the work of Melvin Lax (see^32^ and references therein). The equation of motion for a quantum operator ${\hat{A}}_{\mu }(t)$ subjected to a random Markoffian noise force ${\hat{F}}_{\mu }(t)$ is given as^32^:

28 $$\begin{aligned} \frac{d{\hat{A}}_{\mu }(t)}{dt} = \frac{d\langle {\hat{A}}_{\mu }(t) \rangle }{dt} + {\hat{F}}_{\mu }(t), \end{aligned}$$

where

29 $$\begin{aligned} \langle {\hat{F}}_{\mu }(t) \rangle&= 0 \end{aligned}$$

30 $$\begin{aligned} \langle \hat{F_{\mu }(t) F_{\nu }(\tau )} \rangle&= 2\langle {\hat{D}}_{\mu \nu }(t) \rangle \, \delta (t-\tau ). \end{aligned}$$

For a system with Gaussian noise statistics, the moments in Eqs. (29) and (30) are sufficient to describe the dynamics. Of key importance is the definition of the generalized time-dependent Einstein relation for the diffusion matrix^32,33^ ${\hat{D}}_{\mu \nu }(t)$ relating quantum operators ${\hat{A}}_{\mu }(t)$, ${\hat{A}}_{\nu }(t)$, and $\hat{A_{\mu }(t)A_{\nu }(t)}$:

31 $$\begin{aligned} 2\langle {\hat{D}}_{\mu \nu }(t) \rangle = \frac{d}{dt} \langle \hat{A_{\mu }(t) A_{\nu }(t)} \rangle - \left\langle \frac{d \langle {\hat{A}}_{\mu }(t) \rangle }{dt} {\hat{A}}_{\nu }(t) \right\rangle - \left\langle {\hat{A}}_{\mu }(t) \frac{d \langle {\hat{A}}_{\nu }(t) \rangle }{dt} \right\rangle . \end{aligned}$$

The quantity $2\langle {\hat{D}}_{\mu \nu }(t) \rangle$ in Eq. (31) measures the extent to which the chain rule for differentiating a product has been violated^32^. Since the time-ordered method described above assumes the molecular operators are functions of the fields, it is appropriate to calculate the relationship between the intensity and field operators:

32 $$\begin{aligned} 2\langle {\hat{D}}_{ss} \rangle = \frac{d}{dt} \langle \hat{a^{\dagger }_s a_s }\rangle - \left\langle \frac{d \langle {\hat{a}}^{\dagger }_s \rangle }{dt} {\hat{a}}_s \right\rangle - \left\langle {\hat{a}}^{\dagger }_s \frac{d \langle {\hat{a}}_s \rangle }{dt} \right\rangle , \end{aligned}$$

where

33 $$\begin{aligned} \frac{d}{dt} \langle \hat{a^{\dagger }_s a_s} \rangle&= -i\frac{G}{\hbar } \left[ e^{-i \Delta t} \, \left( {\hat{a}}^{\dagger }_p {\hat{\sigma }}^{(2)}_{ba} + \frac{ \partial {\hat{\sigma }}^{(2)}_{ba}}{\partial {\hat{a}}_p} \right) {\hat{a}}_s + e^{-i \Delta t} \, {\hat{a}}^{\dagger }_p \left( {\hat{\sigma }}^{(1)}_{ba} {\hat{a}}_s + \frac{ \partial {\hat{\sigma }}^{(1)}_{ba}}{\partial {\hat{a}}^{\dagger }_s} \right) - e^{i \Delta t} \, \left( {\hat{a}}^{\dagger }_s {\hat{\sigma }}^{(2)}_{ab} + \frac{ \partial {\hat{\sigma }}^{(2)}_{ab}}{\partial {\hat{a}}_s} \right) {\hat{a}}_p \right. \end{aligned}$$

34 $$\begin{aligned}&\left. - e^{i \Delta t} \, {\hat{a}}^{\dagger }_s \left( {\hat{\sigma }}^{(1)}_{ab} {\hat{a}}_p + \frac{ \partial {\hat{\sigma }}^{(1)}_{ab}}{\partial {\hat{a}}^{\dagger }_p} \right) \right] \end{aligned}$$

35 $$\begin{aligned} \frac{d \langle {\hat{a}}^{\dagger }_s \rangle }{dt}&= -i \frac{G}{\hbar } e^{-i \Delta t} \, \left[ {\hat{a}}^{\dagger }_p {\hat{\sigma }}^{(2)}_{ba} + \frac{ \partial {\hat{\sigma }}^{(2)}_{ba} }{\partial {\hat{a}}_p} + {\hat{a}}^{\dagger }_p {\hat{\sigma }}^{(1)}_{ba} \right] \end{aligned}$$

36 $$\begin{aligned} \frac{d \langle {\hat{a}}_s \rangle }{dt}&= i \frac{G}{\hbar } e^{i \Delta t} \, \left[ {\hat{\sigma }}^{(2)}_{ab} {\hat{a}}_p + {\hat{\sigma }}^{(1)}_{ab} {\hat{a}}_p + \frac{ \partial {\hat{\sigma }}^{(1)}_{ab} }{\partial {\hat{a}}^{\dagger }_p}\right] . \end{aligned}$$

The modified equations of motion for the fields are then

37 $$\begin{aligned} \dot{{\hat{a}}}^{\dagger }_s&= \frac{d \langle {\hat{a}}^{\dagger }_s \rangle }{dt} + {\hat{F}}^{\dagger }_s(t) \end{aligned}$$

38 $$\begin{aligned} \dot{{\hat{a}}}_s&= \frac{d \langle {\hat{a}}_s \rangle }{dt} + {\hat{F}}_s(t) \end{aligned}$$

39 $$\begin{aligned} \langle \hat{F^{\dagger }_s(t) F_s(\tau )} \rangle&= 2 \langle {\hat{D}}_{ss}(t) \rangle \, \delta (t-\tau ) \end{aligned}$$

40 $$\begin{aligned} 2 \langle {\hat{D}}_{ss}(t) \rangle&= -i\frac{G}{\hbar } \left[ e^{-i \Delta t} \, {\hat{a}}^{\dagger }_p \frac{\partial {\hat{\sigma }}^{(1)}_{ba}}{\partial {\hat{a}}^{\dagger }_s} - e^{i \Delta t} \, \frac{ \partial {\hat{\sigma }}^{(2)}_{ab}}{\partial {\hat{a}}_s} {\hat{a}}_p \right] . \end{aligned}$$

Although the above has been calculated for the Stokes field, the same procedure may be applied to the pump field, as well. It may also be applied to the two-level atomic system outlined in the next section in order to characterize the onset of superfluorescence^34–36^.

Of significant importance is the fact that the diffusion matrix relating the intensity to the fields is fully time-dependent and derived from the total system Hamiltonian, unlike in similar approaches^19,31,35,36^. This has great consequences when higher-order interaction terms are included in the Hamiltonian. These higher-order interaction terms may very well become important for ultra-intense laser pulses, specifically of the kind used for stimulated Raman backscattering amplification (SRBA) in plasma^27^, and may affect estimates of parasitic instabilities^37^.

### The two-level atomic system in vacuum

Traditionally, stimulated and spontaneous radiation have been investigated assuming a model two-level atom in which a single electron transitions between upper and lower energy states, and there is a large body of significant, historical literature detailing the development of scientific understanding of this system (see the references listed in the “Introduction”).

The aim of this section is to show in a heuristic manner how the present technique fits into this historical narrative. In particular, it is shown how the Einstein *A* coefficient that characterizes the rate of spontaneous emission may be derived in a straightforward manner that parallels the Weisskopf–Wigner approximation^38^. The Lamb frequency shift is also calculated.

A two-level atom is assumed with electronic ground state ${|}{b}{\rangle }$ and excited state ${|}{a}{\rangle }$, with atomic operators $\hat{\tilde{\sigma }}_{ij}$ that obey the Heisenberg equation of motion. The dynamics of the system are governed by an unperturbed Hamiltonian ${\hat{H}}_A$, an interaction Hamiltonian ${\hat{H}}_I$, and a field Hamiltonian ${\hat{H}}_F$ such that

41 $$\begin{aligned} {\hat{H}} = {\hat{H}}_A + {\hat{H}}_I + {\hat{H}}_F , \end{aligned}$$

where

42 $$\begin{aligned} {\hat{H}}_A&= \hbar \omega _a \, \hat{\tilde{\sigma }}_{aa} + \hbar \omega _b \, \hat{\tilde{\sigma }}_{bb} \end{aligned}$$

43 $$\begin{aligned} {\hat{H}}_I&= - \sum _{ij{\mathbf{k}}} {\mathscr {P}}_{ij{\mathbf{k}}} \, {\mathscr {E}}_{{\mathbf{k}}} \left( \hat{{\tilde{a}}}_{{\mathbf{k}}} + \hat{{\tilde{a}}}^{\dagger }_{{\mathbf{k}}} \right) \, \hat{\tilde{\sigma }}_{ij} \end{aligned}$$

44 $$\begin{aligned} {\hat{H}}_F&= \sum _k \hbar \omega _k \left[ \hat{{\tilde{a}}}_k^{\dagger } \hat{{\tilde{a}}}_k + \frac{1}{2} \right] , \end{aligned}$$

where ${\mathscr {P}}_{ij{\mathbf{k}}} \equiv {\langle }{i}{|} \varvec{\mu }_{ij} \cdot \varvec{\epsilon }_{{\mathbf{k}}} {|}{j}{\rangle }$, $\varvec{\mu }_{ij} \equiv e \, {\mathbf{r}}_{ij}$, ${\mathscr {E}}_{{\mathbf{k}}} \equiv \left( \hbar \omega _{{\mathbf{k}}} / 2 \epsilon _0 V \right) ^{1/2}$, and *V* is the volume of integration over the field mode. Letting $\hat{{\tilde{a}}}_{{\mathbf{k}}} = {\hat{a}}_{{\mathbf{k}}} \exp {\left( -i \omega _{{\mathbf{k}}} t \right) }$ and $\hat{\tilde{\sigma }}_{ij} = {\hat{\sigma }}_{ij} \exp {\left( i \omega _{ij} t \right) }$ and taking the rotating wave approximation, the technique outlined above for the Raman system yields the equations of motion

45 $$\begin{aligned} \dot{{\hat{\sigma }}}_{ab}&= \sum _{{\mathbf{k}}} -i \frac{{\mathscr {P}}_{ba{\mathbf{k}}} \, {\mathscr {E}}_{{\mathbf{k}}}}{\hbar } \, e^{-i\left( \omega _{ab}-\omega _{{\mathbf{k}}} \right) t } \left[ \overset{2}{\overline{ {\hat{a}}^{\dagger }_{{\mathbf{k}}} \, {\hat{\sigma }}_{bb}}} - \overset{1}{\overline{ {\hat{a}}^{\dagger }_{{\mathbf{k}}} \, {\hat{\sigma }}_{aa}} } \right] \end{aligned}$$

46 $$\begin{aligned} \dot{{\hat{\sigma }}}_{ba}&= \sum _{{\mathbf{k}}} -i \frac{{\mathscr {P}}_{ab{\mathbf{k}}} \, {\mathscr {E}}_{{\mathbf{k}}}}{\hbar } \, e^{i\left( \omega _{ab}-\omega _{{\mathbf{k}}} \right) t } \left[ \overset{2}{\overline{ {\hat{a}}_{{\mathbf{k}}} \, {\hat{\sigma }}_{aa}}} - \overset{1}{\overline{ {\hat{a}}_{{\mathbf{k}}} \, {\hat{\sigma }}_{bb}} } \right] \end{aligned}$$

47 $$\begin{aligned} \dot{{\hat{\sigma }}}_{aa}&= \sum _{{\mathbf{k}}} -i \frac{ {\mathscr {E}}_{{\mathbf{k}}}}{\hbar } \, \left[ {\mathscr {P}}_{ba{\mathbf{k}}} \, e^{-i\left( \omega _{ab}-\omega _{{\mathbf{k}}} \right) t } \, \overset{2}{\overline{ {\hat{a}}^{\dagger }_{{\mathbf{k}}} \, {\hat{\sigma }}_{ba}}} - {\mathscr {P}}_{ab{\mathbf{k}}} \, e^{i\left( \omega _{ab}-\omega _{{\mathbf{k}}} \right) t } \, \overset{1}{\overline{ {\hat{a}}_{{\mathbf{k}}} \, {\hat{\sigma }}_{ab}}} \right] \end{aligned}$$

48 $$\begin{aligned} \dot{{\hat{\sigma }}}_{bb}&= \sum _{{\mathbf{k}}} -i \frac{ {\mathscr {E}}_{{\mathbf{k}}}}{\hbar } \, \left[ {\mathscr {P}}_{ab{\mathbf{k}}} \, e^{i\left( \omega _{ab}-\omega _{{\mathbf{k}}} \right) t } \, \overset{2}{\overline{ {\hat{a}}_{{\mathbf{k}}} \, {\hat{\sigma }}_{ab}}} - {\mathscr {P}}_{ba{\mathbf{k}}} \, e^{-i\left( \omega _{ab}-\omega _{{\mathbf{k}}} \right) t } \, \overset{1}{\overline{ {\hat{a}}^{\dagger }_{{\mathbf{k}}} \, {\hat{\sigma }}_{ba}}} \right] \end{aligned}$$

49 $$\begin{aligned} \dot{{\hat{a}}}_{{\mathbf{k}}}&= i \frac{{\mathscr {P}}_{ba{\mathbf{k}}} \, {\mathscr {E}}_{{\mathbf{k}}}}{\hbar } \, \overset{1}{{\hat{\sigma }}}_{ba} \end{aligned}$$

50 $$\begin{aligned} \dot{{\hat{a}}}^{\dagger }_{{\mathbf{k}}}&= -i \frac{{\mathscr {P}}_{ab{\mathbf{k}}} \, {\mathscr {E}}_{{\mathbf{k}}}}{\hbar } \, \overset{1}{{\hat{\sigma }}}_{ab} \end{aligned}$$

51 $$\begin{aligned} \dot{{\hat{n}}}_{{\mathbf{k}}}&= -i \frac{ {\mathscr {E}}_{{\mathbf{k}}}}{\hbar } \, \left[ {\mathscr {P}}_{ab{\mathbf{k}}} \, e^{i\left( \omega _{ab}-\omega _{{\mathbf{k}}} \right) t } \, \overset{2}{\overline{ {\hat{a}}_{{\mathbf{k}}} \, {\hat{\sigma }}_{ab}}} - {\mathscr {P}}_{ba{\mathbf{k}}} \, e^{-i\left( \omega _{ab}-\omega _{{\mathbf{k}}} \right) t } \, \overset{1}{\overline{ {\hat{a}}^{\dagger }_{{\mathbf{k}}} \, {\hat{\sigma }}_{ba}}} \right] , \end{aligned}$$

where ${\hat{n}}_{{\mathbf{k}}} = \hat{a^{\dagger }_{{\mathbf{k}}} a_{{\mathbf{k}}}}$ is the photon number operator. In order to make spontaneous radiative terms explicit in Eqs. (45)–(51), the atom and photon number operators are defined as a combination of two operators, one acting at time-index 1 and the other acting at time-index 2, such that, for example, ${\hat{\sigma }}_{ab} = {\hat{\sigma }}^{(2)}_{ab} + {\hat{\sigma }}^{(1)}_{ab}$. The atom operators are then assumed to be normal-ordered functions of the field operators. Placing all operators in both time- and normal-order then yields the equations of motion

52 $$\begin{aligned} \dot{{\hat{\sigma }}}_{ab}&= \sum _{{\mathbf{k}}} -i \frac{{\mathscr {P}}_{ba{\mathbf{k}}} \, {\mathscr {E}}_{{\mathbf{k}}}}{\hbar } \, e^{-i\left( \omega _{ab}-\omega _{{\mathbf{k}}} \right) t } \left[ {\hat{a}}^{\dagger }_{{\mathbf{k}}} \, \left( {\hat{\sigma }}_{bb} - {\hat{\sigma }}_{aa} \right) + \frac{ \partial }{\partial \, {\hat{a}}_{{\mathbf{k}}}} \left( {\hat{\sigma }}^{(2)}_{bb} - {\hat{\sigma }}^{(2)}_{aa} \right) \right] \end{aligned}$$

53 $$\begin{aligned} \dot{{\hat{\sigma }}}_{ba}&= \sum _{{\mathbf{k}}} -i \frac{{\mathscr {P}}_{ab{\mathbf{k}}} \, {\mathscr {E}}_{{\mathbf{k}}}}{\hbar } \, e^{i\left( \omega _{ab}-\omega _{{\mathbf{k}}} \right) t } \left[ \left( {\hat{\sigma }}_{aa} - {\hat{\sigma }}_{bb} \right) \, {\hat{a}}_{{\mathbf{k}}} + \frac{ \partial }{\partial \, {\hat{a}}^{\dagger }_{{\mathbf{k}}}} \left( {\hat{\sigma }}^{(1)}_{aa} - {\hat{\sigma }}^{(1)}_{bb} \right) \right] \end{aligned}$$

54 $$\begin{aligned} \dot{{\hat{\sigma }}}_{aa}&= \sum _{{\mathbf{k}}} -i \frac{ {\mathscr {E}}_{{\mathbf{k}}}}{\hbar } \, \left[ {\mathscr {P}}_{ba{\mathbf{k}}} \, e^{-i\left( \omega _{ab}-\omega _{{\mathbf{k}}} \right) t } \, \left( {\hat{a}}^{\dagger }_{{\mathbf{k}}} \, {\hat{\sigma }}_{ba} + \frac{\partial \, {\hat{\sigma }}^{(2)}_{ba}}{\partial \, {\hat{a}}_{{\mathbf{k}}}} \right) - {\mathscr {P}}_{ab{\mathbf{k}}} \, e^{i\left( \omega _{ab}-\omega _{{\mathbf{k}}} \right) t } \, \left( {\hat{\sigma }}_{ab} \, {\hat{a}}_{{\mathbf{k}}} + \frac{\partial \, {\hat{\sigma }}^{(1)}_{ab}}{\partial \, {\hat{a}}^{\dagger }_{{\mathbf{k}}}} \right) \right] \end{aligned}$$

55 $$\begin{aligned} \dot{{\hat{\sigma }}}_{bb}&= \sum _{{\mathbf{k}}} -i \frac{ {\mathscr {E}}_{{\mathbf{k}}}}{\hbar } \, \left[ {\mathscr {P}}_{ab{\mathbf{k}}} \, e^{i\left( \omega _{ab}-\omega _{{\mathbf{k}}} \right) t } \, \left( {\hat{\sigma }}_{ab} \, {\hat{a}}_{{\mathbf{k}}} + \frac{\partial \, {\hat{\sigma }}^{(1)}_{ab}}{\partial \, {\hat{a}}^{\dagger }_{{\mathbf{k}}}} \right) - {\mathscr {P}}_{ba{\mathbf{k}}} \, e^{-i\left( \omega _{ab}-\omega _{{\mathbf{k}}} \right) t } \, \left( {\hat{a}}^{\dagger }_{{\mathbf{k}}} \, {\hat{\sigma }}_{ba} + \frac{\partial \, {\hat{\sigma }}^{(2)}_{ba}}{\partial \, {\hat{a}}_{{\mathbf{k}}}} \right) \right] \end{aligned}$$

56 $$\begin{aligned} \dot{{\hat{a}}}_{{\mathbf{k}}}&= i \frac{{\mathscr {P}}_{ba{\mathbf{k}}} \, {\mathscr {E}}_{{\mathbf{k}}}}{\hbar } \, {\hat{\sigma }}_{ba} \end{aligned}$$

57 $$\begin{aligned} \dot{{\hat{a}}}^{\dagger }_{{\mathbf{k}}}&= -i \frac{{\mathscr {P}}_{ab{\mathbf{k}}} \, {\mathscr {E}}_{{\mathbf{k}}}}{\hbar } \, {\hat{\sigma }}_{ab} \end{aligned}$$

58 $$\begin{aligned} \dot{{\hat{n}}}_{{\mathbf{k}}}&= -i \frac{ {\mathscr {E}}_{{\mathbf{k}}}}{\hbar } \, \left[ {\mathscr {P}}_{ab{\mathbf{k}}} \, e^{i\left( \omega _{ab}-\omega _{{\mathbf{k}}} \right) t } \, \left( {\hat{\sigma }}_{ab} \, {\hat{a}}_{{\mathbf{k}}} + \frac{\partial \, {\hat{\sigma }}^{(1)}_{ab}}{\partial \, {\hat{a}}^{\dagger }_{{\mathbf{k}}}} \right) - {\mathscr {P}}_{ba{\mathbf{k}}} \, e^{-i\left( \omega _{ab}-\omega _{{\mathbf{k}}} \right) t } \, \left( {\hat{a}}^{\dagger }_{{\mathbf{k}}} \, {\hat{\sigma }}_{ba} + \frac{\partial \, {\hat{\sigma }}^{(2)}_{ba}}{\partial \, {\hat{a}}_{{\mathbf{k}}}} \right) \right] , \end{aligned}$$

in which all time-indices of the operators have been dropped after having been placed in time- and normal-order. Quantities denoted as type ${\hat{\sigma }}^{(n)}_{ij}$ denote their counterparts in Eqs. (45)–(51).

Equations (52)–(58) may be considerably simplified in the case of an unperturbed atom in the absence of any externally applied fields. Because all of the operators in Eqs. (52)–(58) have been normal-ordered, only their values relative to vacuum are significant. In the absence of an externally applied field and for processes which are entirely spontaneous, the field operators are identically zero and do not change. Physically, it corresponds to the fact that the phase of the spontaneously emitted radiation cannot be predicted. Taking this into account, it is then straightforward to show that the equation of motion for the population of the excited state ${|}{a}{\rangle }$ is given by

59 $$\begin{aligned} \begin{aligned} \dot{{\hat{\sigma }}}_{aa}(t)&= - \sum _{{\mathbf{k}}} \frac{2 \, | {\mathscr {P}}_{ab{\mathbf{k}}} |^2 \, {\mathscr {E}}^2_{{\mathbf{k}}} }{\hbar ^2} \int _0^t \cos {\left[ \left( \omega _{ab} - \omega _{{\mathbf{k}}} \right) \left( t^{\prime } - t \right) \right] \, {\hat{\sigma }}}_{aa}(t^{\prime }) \, dt^{\prime } . \end{aligned} \end{aligned}$$

Assuming closely-spaced field modes,

60 $$\begin{aligned} \sum _{{\mathbf{k}}} \rightarrow 2 \frac{V}{(2 \pi )^3} \int _0^{2\pi } \, d\phi \, \int _0^{\pi } \, \sin {\theta } \, d\theta \, \int _0^{\infty } \, k^2 \, dk , \end{aligned}$$

where the factor of 2 out front accounts for two polarization states. Since $| {\mathscr {P}}_{ab{\mathbf{k}}} |^2 = {\mathscr {P}}_{ab}^2 \, \cos ^2{\theta }$, where $\cos {\theta } = \varvec{\mu }_{ij} \cdot \varvec{\epsilon }_{{\mathbf{k}}}$, Eq. (59) can be written as

61 $$\begin{aligned} \begin{aligned} \dot{{\hat{\sigma }}}_{aa}(t)&= -\frac{1}{4 \pi ^2 \epsilon _0} \frac{4}{3} \frac{ {\mathscr {P}}_{ab}^2}{\hbar c^3} \int _0^{\infty } \int _0^t \omega _{{\mathbf{k}}}^3 \cos {\left[ \left( \omega _{ab} - \omega _{{\mathbf{k}}} \right) \left( t^{\prime } - t \right) \right] \, {\hat{\sigma }}}_{aa}(t^{\prime }) \, dt^{\prime } \, d\omega _{{\mathbf{k}}} . \end{aligned} \end{aligned}$$

Parallel to the Weisskopf–Wigner approximation^38,39^, it can be seen that the time integral is non-negligible only in the region $\omega _{{\mathbf{k}}} = \omega _{ab}$. $\omega _{{\mathbf{k}}}^3$ can therefore be replaced with $\omega _{ab}^3$, and the lower limit on the frequency integral is extended to $-\infty$. Furthermore, since

62 $$\begin{aligned} \int _{-\infty }^{\infty } \cos {\left[ \left( \omega _{ab} - \omega _{{\mathbf{k}}} \right) \left( t^{\prime } - t \right) \right] } \, d\omega _{{\mathbf{k}}} = 2\pi \, \delta (t^{\prime } - t) , \end{aligned}$$

Equation (61) can be written as

63 $$\begin{aligned} \dot{{\hat{\sigma }}}_{aa} = - A \, {\hat{\sigma }}_{aa}, \end{aligned}$$

where

64 $$\begin{aligned} A = \frac{1}{4 \pi \epsilon _0} \frac{4}{3} \frac{ {\mathscr {P}}_{ab}^2 \, \omega _{ab}^3}{\hbar c^3} \, \end{aligned}$$

is the well-known Einstein coefficient that characterizes the rate of spontaneous emission. Following a similar argument, it is straightforward to show that for the population of the ground state

65 $$\begin{aligned} \dot{{\hat{\sigma }}}_{bb} = A \, {\hat{\sigma }}_{aa}, \end{aligned}$$

and therefore

66 $$\begin{aligned} \dot{{\hat{\sigma }}}_{bb} = - \dot{{\hat{\sigma }}}_{aa}. \end{aligned}$$

Equations (63), (65), and (66) show that, while the dynamics of the ground state population depend on the excited state population, the dynamics of the excited state population are independent of the ground state population. Consequently, an atom initially in the excited state can decay spontaneously to the ground state, but an atom initially in the ground state can never spontaneously transition to the excited state. This is in good agreement with energy conservation and all accepted theories of spontaneously emitted radiation^11^.

Finally, a total photon number operator ${\hat{N}}$ can be defined such that

67 $$\begin{aligned} {\hat{N}} = \sum _{{\mathbf{k}}} {\hat{n}}_{{\mathbf{k}}} . \end{aligned}$$

Using Eq. (58), it can easily be shown that

68 $$\begin{aligned} \dot{{\hat{N}}} = \dot{{\hat{\sigma }}}_{bb} , \end{aligned}$$

which again is in agreement with accepted theories^39^.

The Lamb shift can be calculated through analysis of the time-dependence of the atomic de-excitation operator ${\hat{\sigma }}_{ba}$ and takes the form of a slight frequency shift^40^. The equation of motion for the atomic de-excitation operator in the absence of an externally applied field is

69 $$\begin{aligned} \dot{{\hat{\sigma }}}_{ba}(t) = - \sum _{{\mathbf{k}}} \frac{ \, | {\mathscr {P}}_{ab{\mathbf{k}}} |^2 \, {\mathscr {E}}^2_{{\mathbf{k}}} }{\hbar ^2} \int _0^t \exp {\left[ -i\left( \omega _{ab} - \omega _{{\mathbf{k}}} \right) \left( t^{\prime } - t \right) \right] \, {\hat{\sigma }}}_{ba}(t^{\prime }) \, dt^{\prime } . \end{aligned}$$

Since ${\hat{\sigma }}_{ba}$ is assumed to be a slowly varying function of time, it may be taken outside the integral. As time advances^40^,

70 $$\begin{aligned} \lim _{t \rightarrow \infty } \int _0^t \exp {\left[ -i\left( \omega _{ab} - \omega _{{\mathbf{k}}} \right) \left( t^{\prime } - t \right) \right] } \, dt^{\prime } = i \frac{\textit{P}}{\omega _{ab}-\omega _{{\mathbf{k}}}} + \pi \, \delta (\omega _{ab}-\omega _{{\mathbf{k}}}) , \end{aligned}$$

where $\textit{P}$ denotes the principal value. Assuming closely-spaced field modes (as above), Eq. (69) may then be written in the form

71 $$\begin{aligned} \dot{{\hat{\sigma }}}_{ba} = - \left[ i \Lambda + \frac{A}{2} \right] {\hat{\sigma }}_{ba} , \end{aligned}$$

where *A* is the Einstein coefficient defined in Eq. (64) and $\Lambda$ is the Lamb frequency shift given by

72 $$\begin{aligned} \Lambda = \frac{1}{6 \pi ^2 \epsilon _0} \frac{{\mathscr {P}}^2_{ab}}{\hbar c^3} \, \, \textit{P} \int ^{\infty }_0 \frac{\omega ^3}{\omega _{ab}-\omega } \, d\omega , \end{aligned}$$

which is identically equal to accepted estimates^40^. Typically, it is customary to evaluate the integral in Eq. (72) with a high-frequency cutoff^41,42^ of $mc^2/\hbar$.

### The two-level atomic system embedded in a lossy dielectric

Spontaneous radiation in lossy media is an ongoing area of research^43–46^. Here, the simple case of an infinite, homogeneous, and isotropic medium is treated, with the understanding that the interested reader may generalize the approach to any Hamiltonian that sufficiently describes the system under investigation.

The canonical quantization of the electromagnetic field within a dispersive and lossy dielectric medium obeying the Kramers–Kronig relations is performed following the approach of Huttner and Barnett^47^, resulting in boson creation and annihilation operators ${\hat{C}}^{\dagger }$ and ${\hat{C}}$ which correspond to polaritons instead of free-space photons. Significantly, in this approach, the one-to-one relation between the wave vector and frequency of the field in the dielectric is lost: in a lossy dielectric medium, the fields are expressed as continuous double integrals over both the wave vector and the frequency, which are treated as independent variables^47^.

The polariton creation and annihilation operators obey the commutation relation

73 $$\begin{aligned} \left[ {\hat{C}}({\mathbf{k}},\omega ), {\hat{C}}^{\dagger }({\mathbf{k}}^{\prime },\omega ^{\prime }) \right] = \delta \left( {\mathbf{k}}-{\mathbf{k}}^{\prime }\right) \, \delta \left( \omega -\omega ^{\prime }\right) , \end{aligned}$$

and the transverse electric field has the form

74 $$\begin{aligned} \hat{\mathbf{E }}({\mathbf{r}},t) = {\mathscr {E}} \int d^3{\mathbf{k}} \, \, \varvec{\epsilon }_{{\mathbf{k}}} \int ^{\infty }_0 d\omega \, \left[ f({\mathbf{k}},\omega ) \, {\hat{C}}({\mathbf{k}},\omega ) \, e^{-i \left( \omega t - {\mathbf{k}} \cdot {\mathbf{r}} \right) } + \text {adj.}\right] , \end{aligned}$$

where ${\mathscr {E}} \equiv \left( \hbar \omega ^2_c / 2 \epsilon _0 V \right) ^{1/2}$, *V* is the volume of integration over the field mode, $\omega ^2_c = \alpha ^2/\rho \epsilon _0$, $\alpha$ is the charge density, and $\rho$ is the effective mass density associated with the harmonic polarization (see^47,48^ for details). The function $f({\mathbf{k}},\omega )$ is defined as

75 $$\begin{aligned} f({\mathbf{k}},\omega ) = \left( \frac{\epsilon (\omega )+2}{3}\right) \frac{\omega \, \zeta ^*(\omega )}{\omega ^2 \, \epsilon (\omega ) - k^2 c^2 }, \end{aligned}$$

where the dimensionless function $\zeta ({\omega })$ characterizes the frequency dependence of the coupling between the electromagnetic and dressed matter fields. A local field correction factor $(\epsilon +2)/3$ has also been included (consistent with the approach in^48,49^). The local field correction factor can in general be derived from first principles through a more detailed consideration of Umklapp processes^50^. Also, the complex dielectric function $\epsilon = \epsilon ^{\prime } + i \epsilon ^{\prime \prime }$ obeys the Kramers–Kronig relations and is defined as

76 $$\begin{aligned} \epsilon (\omega ) = 1 + \frac{\omega ^2_c}{2\omega } \left[ \textit{P} \, \int _{-\infty }^{\infty } d\omega ^{\prime } \, \frac{ | \zeta (\omega ^{\prime })|^2}{\omega ^{\prime } \left( \omega ^{\prime } - \omega \right) }+ i \pi \frac{ | \zeta (\omega )|^2}{\omega } \right] . \end{aligned}$$

A two-level impurity atom is assumed to be embedded within the medium at the origin and has electronic ground state ${|}{b}{\rangle }$ and excited state ${|}{a}{\rangle }$, with atomic operators $\hat{\tilde{\sigma }}_{ij}$ that obey the Heisenberg equation of motion. The dynamics of the impurity atomic system are governed by an unperturbed Hamiltonian ${\hat{H}}_A$, an interaction Hamiltonian ${\hat{H}}_I$, and a field Hamiltonian ${\hat{H}}_F$ such that

77 $$\begin{aligned} {\hat{H}} = {\hat{H}}_A + {\hat{H}}_I + {\hat{H}}_F , \end{aligned}$$

where

78 $$\begin{aligned} {\hat{H}}_A&= \hbar \omega _a \, \hat{\tilde{\sigma }}_{aa} + \hbar \omega _b \, \hat{\tilde{\sigma }}_{bb} \end{aligned}$$

79 $$\begin{aligned} {\hat{H}}_I&= - {\mathscr {E}} \int d^3{\mathbf{k}} \, {\mathscr {P}}_{ij{\mathbf{k}}} \int ^{\infty }_0 d\omega \, \left[ f({\mathbf{k}},\omega ) \, \hat{{\tilde{C}}}({\mathbf{k}},\omega ) + f^*({\mathbf{k}},\omega ) \, \hat{{\tilde{C}}}^{\dagger }({\mathbf{k}},\omega ) \right] \, \hat{\tilde{\sigma }}_{ij} \end{aligned}$$

80 $$\begin{aligned} {\hat{H}}_F&= \int d^3{\mathbf{k}} \, \int ^{\infty }_0 d\omega \,\, \hbar \omega \,\, \hat{{\tilde{C}}}^{\dagger }({\mathbf{k}},\omega )\, \hat{{\tilde{C}}}({\mathbf{k}},\omega ) , \end{aligned}$$

where ${\mathscr {P}}_{ij{\mathbf{k}}} \equiv {\langle }{i}{|} \varvec{\mu }_{ij} \cdot \varvec{\epsilon }_{{\mathbf{k}}} {|}{j}{\rangle }$, $\varvec{\mu }_{ij} \equiv e \, {\mathbf{r}}_{ij}$, $\hat{{\tilde{C}}}({\mathbf{k}},\omega ) = {\hat{C}}({\mathbf{k}},\omega ) \exp {\left( -i \omega t \right) }$, and $\hat{\tilde{\sigma }}_{ij} = {\hat{\sigma }}_{ij} \exp {\left( i \omega _{ij} t \right) }$. Taking the rotating wave approximation, the equations of motion for the impurity atomic system are

81 $$\begin{aligned} \dot{{\hat{\sigma }}}_{ab}&= -i \frac{{\mathscr {E}}}{\hbar } \, \int d^3{\mathbf{k}} \, {\mathscr {P}}_{ba{\mathbf{k}}} \, \int ^{\infty }_0 d\omega \,\, e^{-i\left( \omega _{ab}-\omega \right) t } \, f^*({\mathbf{k}},\omega ) \left[ \overset{2}{\overline{ {\hat{C}}^{\dagger }({\mathbf{k}},\omega ) \, {\hat{\sigma }}_{bb}}} - \overset{1}{\overline{ {\hat{C}}^{\dagger }({\mathbf{k}},\omega ) \, {\hat{\sigma }}_{aa}} } \right] \end{aligned}$$

82 $$\begin{aligned} \dot{{\hat{\sigma }}}_{ba}&= -i \frac{{\mathscr {E}}}{\hbar } \, \int d^3{\mathbf{k}} \, {\mathscr {P}}_{ab{\mathbf{k}}} \, \int ^{\infty }_0 d\omega \,\, e^{i\left( \omega _{ab}-\omega \right) t } \, f({\mathbf{k}},\omega ) \left[ \overset{2}{\overline{ {\hat{C}}({\mathbf{k}},\omega ) \, {\hat{\sigma }}_{aa}}} - \overset{1}{\overline{ {\hat{C}}({\mathbf{k}},\omega ) \, {\hat{\sigma }}_{bb}} } \right] \end{aligned}$$

83 $$\begin{aligned} \dot{{\hat{\sigma }}}_{aa}&= -i \frac{{\mathscr {E}}}{\hbar } \, \int d^3{\mathbf{k}} \, \int ^{\infty }_0 d\omega \, \left[ {\mathscr {P}}_{ba{\mathbf{k}}} \, e^{-i\left( \omega _{ab}-\omega \right) t } \, f^*({\mathbf{k}},\omega ) \, \overset{2}{\overline{ {\hat{C}}^{\dagger }({\mathbf{k}},\omega ) \, {\hat{\sigma }}_{ba}}} - {\mathscr {P}}_{ab{\mathbf{k}}} \, e^{i\left( \omega _{ab}-\omega \right) t } \, f({\mathbf{k}},\omega ) \, \overset{1}{\overline{ {\hat{C}}({\mathbf{k}},\omega ) \, {\hat{\sigma }}_{ab}}} \right] \end{aligned}$$

84 $$\begin{aligned} \dot{{\hat{\sigma }}}_{bb}&= -i \frac{{\mathscr {E}}}{\hbar } \, \int d^3{\mathbf{k}} \, \int ^{\infty }_0 d\omega \, \left[ {\mathscr {P}}_{ab{\mathbf{k}}} \, e^{i\left( \omega _{ab}-\omega \right) t } \, f({\mathbf{k}},\omega ) \, \overset{2}{\overline{ {\hat{C}}({\mathbf{k}},\omega ) \, {\hat{\sigma }}_{ab}}} - {\mathscr {P}}_{ba{\mathbf{k}}} \, e^{-i\left( \omega _{ab}-\omega \right) t } \, f^*({\mathbf{k}},\omega ) \overset{1}{\overline{ {\hat{C}}^{\dagger }({\mathbf{k}},\omega ) \, {\hat{\sigma }}_{ba}}} \right] . \end{aligned}$$

Again, the impurity atom operators are then assumed to be normal-ordered functions of the field polariton operators. Placing all operators in both time- and normal-order then yields

85 $$\begin{aligned} \dot{{\hat{\sigma }}}_{ab}&= -i \frac{{\mathscr {E}}}{\hbar } \, \int d^3{\mathbf{k}} \, {\mathscr {P}}_{ba{\mathbf{k}}} \, \int ^{\infty }_0 d\omega \,\, e^{-i\left( \omega _{ab}-\omega \right) t } \, f^*({\mathbf{k}},\omega ) \left[ {\hat{C}}^{\dagger }({\mathbf{k}},\omega ) \, \left( {\hat{\sigma }}_{bb} - {\hat{\sigma }}_{aa} \right) + \frac{ \partial }{\partial \, {\hat{C}}({\mathbf{k}},\omega )} \left( {\hat{\sigma }}^{(2)}_{bb} - {\hat{\sigma }}^{(2)}_{aa} \right) \right] \end{aligned}$$

86 $$\begin{aligned} \dot{{\hat{\sigma }}}_{ba}&= -i \frac{{\mathscr {E}}}{\hbar } \, \int d^3{\mathbf{k}} \, {\mathscr {P}}_{ab{\mathbf{k}}} \, \int ^{\infty }_0 d\omega \,\, e^{i\left( \omega _{ab}-\omega \right) t } \, f({\mathbf{k}},\omega ) \left[ \left( {\hat{\sigma }}_{aa} - {\hat{\sigma }}_{bb} \right) \, {\hat{C}}({\mathbf{k}},\omega ) + \frac{ \partial }{\partial \, {\hat{C}}^{\dagger }({\mathbf{k}},\omega )} \left( {\hat{\sigma }}^{(1)}_{aa} - {\hat{\sigma }}^{(1)}_{bb} \right) \right] \end{aligned}$$

87 $$\begin{aligned} \dot{{\hat{\sigma }}}_{aa}&= -i \frac{{\mathscr {E}}}{\hbar } \, \int d^3{\mathbf{k}} \, \int ^{\infty }_0 d\omega \, \left[ {\mathscr {P}}_{ba{\mathbf{k}}} \, e^{-i\left( \omega _{ab}-\omega \right) t } \, f^*({\mathbf{k}},\omega ) \left( {\hat{C}}^{\dagger }({\mathbf{k}},\omega ) \, {\hat{\sigma }}_{ba} + \frac{\partial \, {\hat{\sigma }}^{(2)}_{ba}}{\partial \, {\hat{C}}({\mathbf{k}},\omega )} \right) \right. \end{aligned}$$

88 $$\begin{aligned}&- \left. {\mathscr {P}}_{ab{\mathbf{k}}} \, e^{i\left( \omega _{ab}-\omega \right) t } \, f({\mathbf{k}},\omega ) \left( {\hat{\sigma }}_{ab} \, {\hat{C}}({\mathbf{k}},\omega ) + \frac{\partial \, {\hat{\sigma }}^{(1)}_{ab}}{\partial \, {\hat{C}}^{\dagger }({\mathbf{k}},\omega )} \right) \right] \end{aligned}$$

89 $$\begin{aligned} \dot{{\hat{\sigma }}}_{bb}&= -i \frac{{\mathscr {E}}}{\hbar } \, \int d^3{\mathbf{k}} \, \int ^{\infty }_0 d\omega \, \left[ {\mathscr {P}}_{ab{\mathbf{k}}} \, e^{i\left( \omega _{ab}-\omega \right) t } \, f({\mathbf{k}},\omega ) \left( {\hat{\sigma }}_{ab} \, {\hat{C}}({\mathbf{k}},\omega ) + \frac{\partial \, {\hat{\sigma }}^{(1)}_{ab}}{\partial \, {\hat{C}}^{\dagger }({\mathbf{k}},\omega )} \right) \right. \end{aligned}$$

90 $$\begin{aligned}&- \left. {\mathscr {P}}_{ba{\mathbf{k}}} \, e^{-i\left( \omega _{ab}-\omega \right) t } \, f^*({\mathbf{k}},\omega ) \left( {\hat{C}}^{\dagger }({\mathbf{k}},\omega ) \, {\hat{\sigma }}_{ba} + \frac{\partial \, {\hat{\sigma }}^{(2)}_{ba}}{\partial \, {\hat{C}}({\mathbf{k}},\omega )} \right) \right] . \end{aligned}$$

Quantities denoted as type ${\hat{\sigma }}^{(n)}_{ij}$ denote their counterparts in Eqs. (81)–(84).

Following the same arguments for an atom in vacuum, the equation of motion for the population of the impurity atomic excited state ${|}{a}{\rangle }$ is given by

91 $$\begin{aligned} \dot{{\hat{\sigma }}}_{aa} = -{\hat{\sigma }}_{aa} \,\, A \,\, \frac{2 \, \omega _{ab} \, c^3 \, \epsilon ^{\prime \prime }(\omega _{ab})}{\pi } \bigg | \frac{\epsilon (\omega _{ab})+2}{3} \bigg |^2 \int _0^{\infty } \frac{k^2 \, \, dk}{\left[ \omega ^2_{ab} \, \epsilon ^{\prime }(\omega _{ab}) - k^2 \, c^2 \right] ^2 + \left[ \omega ^2_{ab} \, \epsilon ^{\prime \prime }(\omega _{ab}) \right] ^2} , \end{aligned}$$

where *A* is the Einstein coefficient for spontaneous decay in vacuum defined in Eq. (64) and the definitions in Eqs. (75) and (76) have been taken into account. The integral in Eq. (91) may be evaluated by contour integration. Defining the real and imaginary parts of the complex refractive index ${\tilde{n}} = n+i\kappa$ as

92 $$\begin{aligned} \epsilon (\omega ) = \epsilon ^{\prime }(\omega )+i\epsilon ^{\prime \prime }(\omega ) = \left[ n(\omega ) +i \kappa (\omega ) \right] ^2. \end{aligned}$$

Equation (91) may be written in form

93 $$\begin{aligned} \dot{{\hat{\sigma }}}_{aa} = -A_L {\hat{\sigma }}_{aa}, \end{aligned}$$

where the modified spontaneous decay rate $A_L$ in a lossy, dispersive dielectric medium is

94 $$\begin{aligned} A_L = A \,\, n(\omega _{ab}) \, \bigg | \frac{\epsilon (\omega _{ab})+2}{3} \bigg |^2 , \end{aligned}$$

which is identically equal to accepted values^48^.

The Lamb shift can also be calculated in an analogous manner to the case of an atom in vacuum. The equation of motion for the atomic de-excitation operator in the absence of an externally applied polariton field is

95 $$\begin{aligned} \dot{{\hat{\sigma }}}_{ba} = - \left[ i \Lambda _L + \frac{A_L}{2} \right] {\hat{\sigma }}_{ba}, \end{aligned}$$

where $\Lambda _L$ is the modified Lamb frequency shift given by

96 $$\begin{aligned} \Lambda _L = \frac{1}{3 \pi ^3 \epsilon _0} \frac{{\mathscr {P}}^2_{ab} }{\hbar } \, \, \, \textit{P} \int _0^{\infty } dk \, \, \int ^{\infty }_0 d\omega \, \, \frac{k^2 \, \, \omega ^4 \, \, \epsilon ^{\prime \prime }(\omega ) }{\left[ \left[ \omega ^2 \, \epsilon ^{\prime }(\omega ) - k^2 \, c^2 \right] ^2 + \left[ \omega ^2 \, \epsilon ^{\prime \prime }(\omega ) \right] ^2 \right] \left[ \omega _{ab}-\omega \right] } \, \, \bigg | \frac{\epsilon (\omega )+2}{3} \bigg |^2 . \end{aligned}$$

## Discussion and conclusion

It may be instructive to discuss the above method in view of the widely used techniques outlined by Shaul Mukamel that calculate contributions to spontaneous radiative processes, as described in^51^. In the latter approach, the calculation of the spontaneous light emission signal mirrors the calculation for the nonlinear response function $S^{(3)}(t_3,t_2,t_1)$, which itself results from a perturbative expansion of the density matrix operator ${\hat{\rho }}(t)$ according to the U-matrix method. The difference in the case of spontaneous radiation is that the emitted field has no photons initially (it is a vacuum state) and therefore must be treated quantum-mechanically instead of classically, as is commonly done in evaluating $S^{(3)}(t_3,t_2,t_1)$. In practice this involves evaluating the action of field operators on field modes, for example ${\hat{a}}^{\dagger }{|}{n}{\rangle } = \sqrt{n+1}{|}{n+1}{\rangle }$ and ${\hat{a}}{|}{n}{\rangle }=\sqrt{n}{|}{n-1}{\rangle }$. Such operations are the origin, mathematically, of the spontaneous radiation in the U-matrix method, as well. Specifically, it is the $+1$ term in $\sqrt{n+1}$ that corresponds to spontaneous radiation^52^. In fact, this $+1$ term results directly from the evaluation of the commutator $\left[ {\hat{a}}, {\hat{a}}^{\dagger } \right]$, such that it is rigorously correct to state that ${\hat{a}}^{\dagger }{|}{n}{\rangle } = \sqrt{n+\left[ {\hat{a}},{\hat{a}}^{\dagger }\right] }{|}{n+1}{\rangle }$ (see Sect. 2.3 of^53^).

Mathematically, then, spontaneous radiation results directly from the noncommutativity of the field creation and annihilation operators. In order to introduce spontaneous radiation into a physical theory, evaluation of field commutators must be correctly implemented. Here, in this work, instead of calculating the action of operators on field modes as in^51^, evaluation of commutators occurs in normal-ordering of time-ordered operator products. The advantage of working directly with the time-ordered Heisenberg equation of motion is that its validity for a given Hamiltonian remains valid for all times. Current widely-used approaches that use a perturbative expansion “may be valid at short times but will always break down at longer times”^51^, and this is clearly shown in Fig. 2. This is not to say that, in many circumstances, the power and versatility of such perturbative approaches (and their variants^54,55^) may not prove to be more convenient and of sufficient accuracy for small *t*.

It should be noted that after the initial expression of the equations of motion in Eqs. (11)–(16), (45)–(51), and (81)–(84), it is assumed that the chemical (either molecule or atom) operators are functions of the field operators and therefore do not commute with them. Conventionally, it is assumed without nuance that the chemical and field operators commute with each other^56^. This is not the case, however, for the chemical’s interaction with spontaneously emitted or scattered radiation. On the contrary, it has been shown that as time progresses, the chemical source operators that give rise to the spontaneous radiation evolve into the joint chemical/field Hilbert space^56,57^, and thus fail to commute. In this sense, it is not surprising that in Eqs. (11)–(16), (45)–(51), and (81)–(84), only the spontaneous terms are affected by an assumption of non-commutativity between the chemical and field operators, while the coherent terms remain unaffected.

In addition, it has long been known that, in the case where chemical operators initially commute, the complementary contributions of radiation reaction and vacuum fluctuations to spontaneous radiative processes can be apportioned in differing amounts by changing the order of the operators. Consequently^7^, it is possible, for example, to express the total Lamb frequency shift $\Lambda$ as a sum of contributions from both radiation reaction (*RR*) and vacuum fluctuations (*VF*) such that $\Lambda = \Lambda _{RR}+\Lambda _{VF}$. In the case where the operators are normal-ordered, $\Lambda _{RR} = \Lambda$ and $\Lambda _{VF} = 0$. When the operators are anti-normal-ordered, $\Lambda _{RR} = -\Lambda$ while $\Lambda _{VF} = 2\Lambda$. In the case of symmetric-ordering, $\Lambda _{RR} = 0$ and $\Lambda _{VF} = \Lambda$. Again, assuming the chemical and field operators initially commute, it is possible to shuffle between these different orderings to emphasize various physical interpretations^7^.

In the method described above, however, the chemical and field operators do not commute, because the chemical operators are assumed to be functions of the fields. This assumption is necessary in order to make use of the initial conditions ${\hat{\sigma }}_{aa}(0)$, ${\hat{\sigma }}_{bb}(0)$. Doing so effectively restricts the ordering of operators to normal-order, since in this context anti-normal ordering yields an excited state population ${\hat{\sigma }}_{aa}$ that is dependent on the ground state population ${\hat{\sigma }}_{bb}$, which is unphysical. In normal-ordering, only radiation reaction contributes to spontaneous radiation. In anti-normal-ordering, both radiation reaction and vacuum fluctuations contribute to spontaneous radiation. In symmetric-ordering, spontaneous radiation is entirely due to vacuum fluctuations, and therefore it is expected that, like anti-normal-ordering, symmetric-ordering will also yield unphysical results. As such, the method outlined in this work attributes spontaneous radiation entirely to radiation reaction.

In the absence of freedom to choose the ordering of chemical and field operators, however, the method described above allows a freedom in the temporal extension of the operators. For example, the convention of initially placing the chemical operators to the right of all the field operators has been used, and as a result the temporal extension of the chemical operators is forward in time. However, equivalent results may also be obtained if the chemical operators are initially placed to the left of all the field operators, and the temporal extension of the chemical operators is backward in time:

97 $$\begin{aligned} \overset{1}{{\hat{a}}_{{\mathbf{k}}}}\left[ \overset{2}{ {\hat{\sigma }}^{(2)}_{ij} } + \overset{1}{ {\hat{\sigma }}^{(1)}_{ij} } \right] = \left[ \overset{1}{ {\hat{\sigma }}^{(2)}_{ij} } + \overset{0}{ {\hat{\sigma }}^{(1)}_{ij} } \right] \overset{1}{{\hat{a}}_{{\mathbf{k}}}}. \end{aligned}$$

Either of the options in Eq. (97) may be used to yield physically meaningful results, as long as the chemical operators are assumed to be normal-ordered functions of the field operators.

In conclusion, it is evident from the above that the Heisenberg equation of motion can account for spontaneous radiative processes in a straightforward way when both time- and normal-ordering of the operators are taken into account. Moreover, this is done within the context of a unified set of partial differential equations, which are amenable to pulse propagation calculations and simulations. While the technique is immediately useful for investigating spontaneous radiative processes in lossy media as well as modeling the interaction between spontaneous and stimulated Raman scattering from high-intensity laser pulses, its simplicity and generality suggest that it may yield fruitful results in many other areas of physics.
